# Depression and Anxiety Are Associated with Physical Performance in Patients Undergoing Cardiac Rehabilitation: A Retrospective Observational Study

**DOI:** 10.3390/jcdd9010021

**Published:** 2022-01-11

**Authors:** Maaya Sakamoto, Yasunori Suematsu, Yuiko Yano, Koji Kaino, Reiko Teshima, Takuro Matsuda, Masaomi Fujita, Rie Tazawa, Kanta Fujimi, Shin-ichiro Miura

**Affiliations:** 1 Department of Cardiology, School of Medicine, Fukuoka University, Fukuoka 814-0180, Japan; motomakas48@gmail.com (M.S.); ysuematsu@fukuoka-u.ac.jp (Y.S.); yuicom0109@gmail.com (Y.Y.); kanta2345@yahoo.co.jp (K.F.); 2 Center for Cardiac Rehabilitation, Fukuoka University Hospital, Fukuoka 814-0180, Japan; goeminence@yahoo.co.jp (K.K.); reiko.dai.222@icloud.com (R.T.); gd050010@yahoo.co.jp (T.M.); omiomi1973@gmail.com (M.F.); 3 Division of Nutrition, Fukuoka University Hospital, Fukuoka 814-0180, Japan; tazawa0220@fukuoka-u.ac.jp; 4 Department of Cardiology, Fukuoka University Nishijin Hospital, Fukuoka 814-8522, Japan

**Keywords:** psychological condition, functional performance, cardiac rehabilitation, ischemic heart disease

## Abstract

Background: Cardiac rehabilitation (CR) combined with stress management training has been shown to be associated with fewer clinical events than CR alone. However, there have been no reports on the associations of CR with the psychological condition and detailed physical activities evaluated on the same day. Method: One hundred outpatients who participated in a CR program were graded on the hospital anxiety and depression scale (HADS). We divided them into a high HADS group (*n* = 32) and a normal HADS group (*n* = 68) and investigated by whole patients, ischemic heart disease (IHD) patients, and heart failure patients. Results: Overall, the patient age was 70.5 ± 9.6 years, the percentage of males was 73.0%, and the body mass index was 23.4 (21.7–26.0) kg/m^2^. In the high HADS group, overall functional mobility was poor and the distance in a two-minute walking test was short. Especially in IHD patients, the high HADS group showed high fat mass in body composition and low exercise tolerance and ventilator equivalents in cardiopulmonary exercise test. Conclusions: Depression and anxiety involved poor physical performance in CR outpatients and particularly involved low exercise tolerance in IHD patients. To evaluate accurate physical performance, it is necessary to investigate psychological condition.

## 1. Introduction

Cardiac rehabilitation (CR) is a program for cardiovascular patients to improve their cardiovascular prognosis, quality of life, and activities of daily living. Mild cognitive impairment is very common in post-acute coronary syndrome patients and makes worse symptoms of depression or anxiety [1]. Exercise-based CR itself improves the psychological state, and completion of a 12-month CR program was associated with a significant reduction of clinical psychological distress [2]. In another study, early 3-week CR for patients who had undergone coronary artery bypass grafting was effective to improve state anxiety for patients with mild anxiety-depressive symptoms, but not severe anxiety-depressive symptoms [3]. The effect of exercise-based CR itself might depend on the severity of patients’ psychological condition. CR combined with stress management training has been shown to be associated with fewer clinical events than CR alone [4]. However, the effect of any specific psychological intervention in CR is controversial. A meta-analysis showed that the effects of specific psychological interventions in CR patients were not general, and therefore these should be focused on patients and conditions where they will be effective [5]. Anxiety and depression are associated with quality of life and are risk factors for the development of cardiovascular diseases [6,7]. To determine the effect of CR combined with psychological intervention, we should know which physical performances are actually associated with anxiety and depression. Psychological condition changes from day to day. To determine accurate association with psychological condition and physical performance, it is better to check both on the same day. However, there have been few investigations of anxiety, depression, and detailed physical performance tests investigated on the same day. Therefore, in this study, we investigated the associations of anxiety and depression with the results of detailed physical performance tests in outpatients undergoing CR.

## 2. Materials and Methods

### 2.1. Study Design

The procedures were performed in accordance with the Declaration of Helsinki and the ethical standards of the Independent Review Board of Fukuoka University. This investigation was approved by the Independent Review Board of Fukuoka University (2018M039).

This study is a retrospective observational study. Outpatients who gave informed consent and performed all tests on the same day were included. Patients were in late recovery period or maintenance period. We excluded the patients who could not perform cardiopulmonary exercise test (CPET). One hundred outpatients who participated in a CR program were graded on the hospital anxiety and depression scale (HADS) and performed detailed physical performance tests on the same day between November 2018 and April 2020.

We checked patient’s backgrounds, frequency of weekly CR participation, comorbidities, basic cardiovascular diseases, and treatments. Patient’s backgrounds were investigated including the patient’s age, sex, body mass index, and left ventricular ejection fraction. Comorbidities were investigated including hypertension, diabetes mellitus, dyslipidemia, chronic kidney disease, chronic obstructive pulmonary disease, other pulmonary disease, and psychiatric disorder. Basic cardiovascular diseases were investigated including ischemic heart disease (IHD), cardiomyopathy, heart failure (HF), valvular disease, and macrovascular disease. Treatments were investigated including percutaneous coronary intervention, coronary artery bypass grafting, valve operation, and implantation of implantable cardioverter-defibrillator and cardiac resynchronization therapy.

We performed body composition and various physical performance tests including the timed up-and-go test (TUG), short physical performance battery (SPPB), one-leg standing time with eyes open, 2 min walking test (2MWT), and a CPET on the same day.

### 2.2. HADS

HADS is a self-rating, patient-reported outcome measure of anxiety and depression [7]. Fourteen items that are equally divided between anxiety and depression are presented on a 4-point Likert scale (range 0–3). The total score is 0 to 7 for normal or no anxiety/depression, 8 to 10 for mild, 11 to14 for moderate, and 15 to 21 for severe anxiety/depression [8]. Cronbach’s alpha score was calculated after examining the forms filled in 14 items. An alpha score of 0.85–0.87 was obtained. The estimated Cronbach’s alpha was higher than 0.85 for all items. We divided the 100 participants into a normal HADS group, in which the scores for both HADS-anxiety and HADS-depression were no more than 7 points, and a high HADS group, in which the score for either HADS-anxiety or HADS-depression was not less than 8 points.

### 2.3. Body Composition

Body composition was checked using an MC-190 (TANITA, Tokyo, Japan). Body weight, lean body mass, body fat mass, percent body fat, dry lean mass, total body water, estimated bone mass, basal metabolic rate, and lean mass and fat mass at the trunk, left arm, right arm, left leg, and right leg were measured.

### 2.4. Physical Performance Tests

We performed TUG, SPPB, one-leg standing time with eyes open test, 2MWT, and CPET.

TUG evaluates overall functional mobility [9,10,11]. Patients stood up from an armchair with a back, walked 3 m, turned around, walked back to the chair, and sat down, and we measured the time. The one-leg standing time with eyes open test was performed with the hands on the waist and we recorded the time until the subject lost balance, moved their hands from their waist, or touched the ground with their raised foot [12]. Patients performed the both-legs test and we measured the time, with a maximum score of 60 s. SPPB is designed to measure functional status and physical performance [13]. SPPB consists of three tests including the standing balance test (together side-by-side, semi-tandem and tandem), 4 m gait test, and chair-stand test. Each score ranged from 0 to 4 and the sum final SPPB score ranged from 0 to 12. A higher score indicates a higher level of function.

It is difficult for some cardiovascular patients to walk for a long time. The 2MWT is suitable for patients with reduced exercise tolerance such as those with respiratory disease [14] or who have undergone cardiac surgery [15]. The 2MWT can provide information similar to that obtained in the 6-min walking test [16]. The 2MWT evaluates heart rate, saturation of percutaneous oxygen, and rate of perceived exertion at baseline, 1 min later, and 2 min later, in addition to walking distance.

### 2.5. CPET

The patients underwent a CPET using Cpex1 (Inter Riha, Tokyo, Japan) and a stress test system with a Strength Ergo 8 (Fukuda Denshi, Tokyo, Japan). We evaluated heart rate, systolic blood pressure, diastolic blood pressure, oxygen uptake per weight (VO_2_/wt), respiratory exchange ratio (R), ventilator equivalents, tidal volume, and respiratory rate at rest, warmup, 1 min before anaerobic threshold (AT), AT, peak, and end periods. Ventilator equivalent versus carbon dioxide output slope, minimum ventilator equivalents for carbon dioxide, oxygen-uptake efficiency slope, the ratio of oxygen uptake increase to work rate increase, and the ratio of heart rate increase to work rate increase were also evaluated.

### 2.6. Statistical Analysis

All data analyses were performed using the SAS (Statistical Analysis System) Software Package (Ver. 9.4, SAS Institute Inc., Cary, NC, USA) at Fukuoka University (Fukuoka, Japan). We analyzed the differences between normal HADS group and high HADS group in whole patients, IHD patients, and HF patients. The patients who had both IHD and HF were excluded from the analysis in IHD patients or HF patients. Continuous variables were expressed as mean ± standard deviation and compared between the groups by Student’s *t*-test. Continuous variables with a non-normal distribution were expressed as median (interquartile range) and compared between groups by the Wilcoxon rank sum test. Categorical variables were compared between the groups by a Chi-square analysis. A value of *p* < 0.05 was considered significant.

## 3. Results

### 3.1. Patient Background

**Figure 1:**
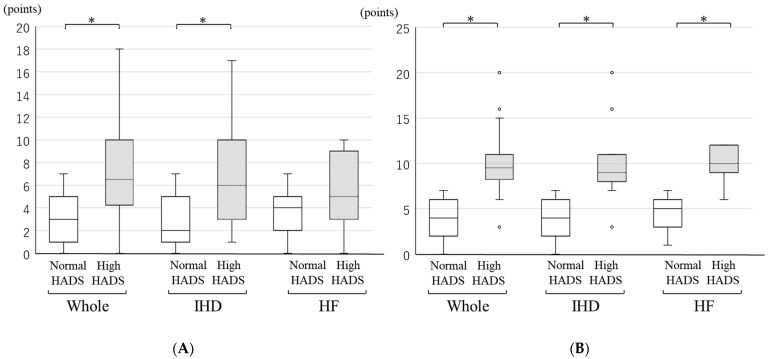
Scores for anxiety and depression by HADS in normal HADS group and high HADS group by whole patients, IHD patients, or HF patients. The scores for (**A**) anxiety and (**B**) depression by HADS in the normal and high HADS group of whole, IHD, and HF patients are shown by a box plot. White box indicates the normal HADS group and grey box indicates the high HADS group. White circle indicates outlier. IHD: ischemic heart disease, HF: heart failure. * *p* < 0.05.

In whole patients, the normal HADS group (*n* = 68) had an anxiety score of 3.0 (1.0–5.0) and a depression score of 4.0 (2.0–6.0). The high HADS group (*n* = 32) had an anxiety score of 6.5 (4.5–10.0) and a depression score of 9.5 (8.5–11.0); both scores in the high HADS group were significantly higher than those in the normal HADS group (Figure 1). In the analyses of IHD and HF patients, the results were similar to the analyses of whole patients. The overall average age, percentage male, average BMI, and average LVEF were 70.5 ± 9.6 years, 73.0%, 23.4 (21.7–26.0) kg/m^2^, and 60.9 (47.5–68.0) %, respectively (Table 1). Patients participated in CR 1.0 (1.0–2.0) times per week. The rates of diabetes mellitus (*p* = 0.02) and dyslipidemia (*p* = 0.02) in the high HADS group were high compared to those in the normal HADS group. There were no significant differences in pulmonary disease, psychiatric disorder, cardiovascular disease, or treatment for cardiovascular disease between the groups.

### 3.2. Body Composition

The overall average body weight, body fat percentage, dry lean mass and basal metabolic rate were 63.2 (55.4–67.5) kg, 25.5 ± 8.0 %, 44.4 ± 8.5 kg, and 1273 ± 191 kcal, respectively, and there were no significant differences in these parameters between the groups in whole patients (Table 2). There were no significant differences in lean mass or fat mass for any of the body parts between the groups in whole patients. The results in HF patients also did not show significant differences between the groups. However, the percent body fat, body fat mass, and the fat mass in any parts were significantly high in the high HADS group of IHD patients.

### 3.3. Physical Performances

We checked TUG, one-leg standing time with eyes open test, and SPPB (Table 3). In whole patients, TUG in the high HADS group was significantly slower than that in the normal HADS group (*p* = 0.01). There were no significant differences in either the one-leg standing time with eyes open test or SPPB between the groups. In the analyses of IHD and HF patients, there were no significant differences.

In the 2MWT, the walking distance in the high HADS group was significantly shorter than that in the normal HADS group (*p* = 0.01). In HF patients, the saturation of percutaneous oxygen after 2 min was significantly low in the high HADS group (Table 4).

### 3.4. CPET

**Figure 2:**
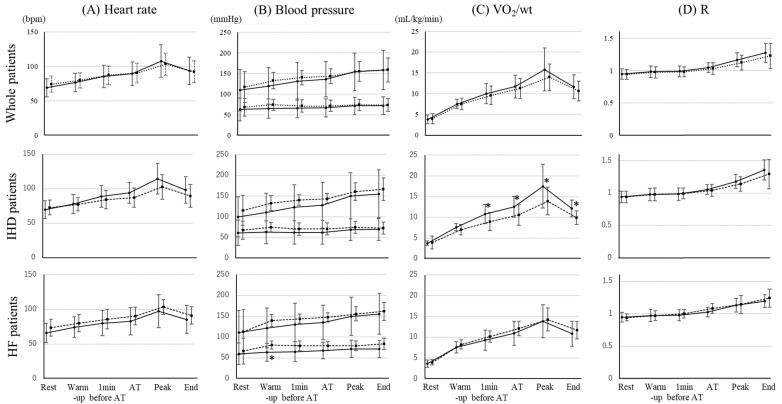
The differences of hemodynamics and exercise tolerance in CPET between normal HADS group and high HADS group in whole patients, IHD patients, and HF patients. (**A**) Heart rate, (**B**) blood pressure, (**C**) VO_2_/wt, and (**D**) respiratory exchange ratio in whole, IHD, and HF patients are shown. A solid line and circle marker indicates the normal HADS group and a dotted line and square marker indicates the high HADS group. CPET: cardiopulmonary exercise test, IHD: ischemic heart disease, HF: heart failure, VO2/Wt: oxygen uptake per weight, R: respiratory exchange ratio, and AT: anaerobic threshold. * *p* < 0.05.

**Figure 3:**
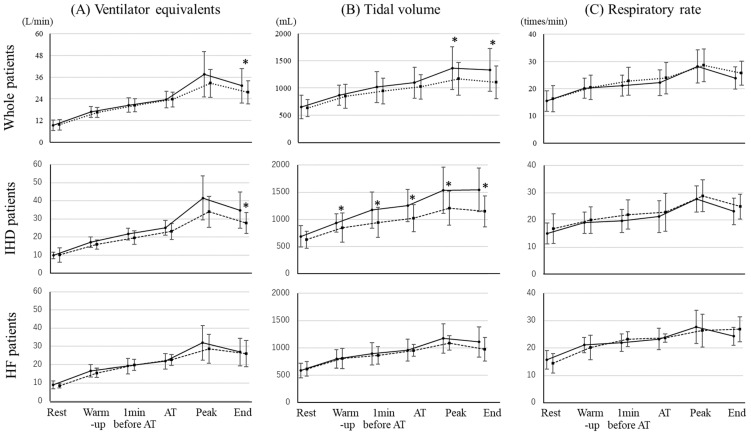
The differences of respiration activities in CPET between normal HADS group and high HADS group in whole patients, IHD patients, and HF patients. (**A**) Ventilator equivalents, (**B**) tidal volume, and (**C**) respiratory rate in whole, IHD, and HF patients are shown. A solid line and circle marker indicates the normal HADS group and a dotted line and square marker indicates the high HADS group. CPET: cardiopulmonary exercise test, IHD: ischemic heart disease, HF: heart failure, AT: anaerobic threshold. * *p* < 0.05.

Blood pressure and heart rate were not significantly different between the groups during CPET in whole, IHD, and HF patients (Figure 2). There was no significant difference in VO_2_/wt between the groups under load conditions, whereas the R was increased in both groups in whole and HF patients. However, VO_2_/wt in the high HADS group of IHD patients was significantly lower from 1 min before AT period. With regard to respiratory activity, tidal volume significantly decreased after the peak period and ventilator equivalents significantly decreased at the end period in the high HADS group of whole patients (Figure 3). In IHD patients, tidal volume in the high HADS group was significantly lower from warmup period and ventilator equivalents were significantly lower at the end period.

In whole patients, the ventilator equivalent versus carbon dioxide output slope (normal HADS group: 28.8 (26.4–34.3), high HADS group: 29.3 (25.5–33.5), *p* = 0.7), minimum ventilator equivalents for carbon dioxide (29.5 ± 6.0 mL/mL, 29.4 ± 5.9 mL/mL, *p* = 0.9), oxygen-uptake efficiency slope (1356 (1107–1868), 1359 (1166–1708), *p* = 0.7), ratio of oxygen uptake increase to work rate increase (8.15 ± 2.12 mL/min/watt, 7.83 ± 1.85 mL/min/watt, *p* = 0.5), and ratio of heart rate increase to work rate increase (0.48 (0.35–0.66) beats/min/watt, 0.44 (0.34–0.50) beats/min/watt, *p* = 0.06) were also evaluated, however there were no significant differences between the groups. In IHD and HF patients, these parameters also did not show significant differences between the groups.

## 4. Discussion

In this study, we evaluated the associations between HADS and detailed physical examinations in CR outpatients. The high HADS group showed poor outcomes with regard to overall functional mobility and walking distance. Especially in IHD patients, the high HADS group showed high fat mass in body consumption and low exercise tolerance and ventilator equivalents in CPET. Depression and anxiety associated with physical performances in patients undergoing cardiac rehabilitation and the association requires consideration of underlying heart disease.

CR is an interdisciplinary approach that involves a medical doctor, nurse, pharmacist, physical therapist, registered dietitian, and clinical psychologist [17]. Recently, psychological intervention in combination with CR has been researched actively. In Denmark, therapist-assisted eHealth intervention targeting depression and anxiety in patients with ischemic heart disease is ongoing [18]. Endurance training together with CR improved physical work capacity [19]. The addition of metacognitive therapy to CR did not have a negative impact on CR in an internal pilot study [20]. In this situation, to establish evidence regarding psychological intervention with exercise-based CR, we must identify which physical parameters are closely associated with the psychological condition. We examined the association between the psychological condition and detailed physical performances. We were able to investigate these associations because the examinations were performed on the same day.

Depression and anxiety are essential parts of psychological condition for care in CR patients [21,22,23,24]. HADS is an efficient method for screening for anxiety and depression in CR patients [21,24]. In this study, instrument-defined anxiety and depression were 14% (*n* = 14) and 29% (*n* = 29), respectively. We did not include any severe symptomatic patients who would have needed additional psychological treatment. The scores of HADS-anxiety and -depression were similar in whole, IHD, and HF patients. In the high HADS group of whole patients, the percentages of diabetes mellitus and dyslipidemia were high. This could reflect the fact that anxiety and depression cause overeating [25] and a high prevalence of diabetes mellitus [26].

There were no significant differences in body composition between the high HADS group and the normal HADS group in whole patients. The results of body composition in IHD patients were different from HF patients. The high HADS group showed high percent body fat, body fat mass, and fat mass in any parts in IHD patients, but not HF patients. These results might involve fear of exercise in IHD patients. IHD patients typically have had an exertional chest pain and felt a fear for exercise. The fear in the high HADS group of IHD patients might restrict effective exercise for reducing body fat.

In CPET, ventilator equivalents at the end of period in the high HADS group were significantly lower than that in the normal HADS group in whole patients. Low ventilator equivalent led to low tidal volume. High anxiety causes low tidal volume [27]. In other words, poor progression of ventilator equivalents during CPET would reveal anxiety and depression. The results of CPET in IHD patients were quite different from HF patients. In IHD patients, the high HADS group showed lower VO_2_/wt than that in the normal HADS group from 1 min before AT period. This result also might associate with a fear for strong exercise in the high HADS group of IHD patients. Low ventilator equivalents due to low tidal volume might involve low peak VO_2_/wt in the high HADS group of IHD patients. As another cause, low physical strength might be simply associated with low peak VO2/wt in the high HADS group of IHD patients.

TUG evaluates overall functional mobility [9,10,11]. The high HADS group showed a long TUG time compared to that in the normal HADS group in whole patients. This could reflect the effect of physical performance on anxiety and depression. In 2MWT, the walking distance in the high HADS group was shorter than that in the normal HADS group in whole patients. In HF patients, the saturation of percutaneous oxygen after 2 min were worsened in the high HADS group. The TUG and 2MWT are suitable for evaluating physical performance to check the effect of psychological intervention.

This study has several limitations. First, the sample size might be small. However, it should be large enough for this study, since some studies on the mental status in CR patients have had even smaller sample sizes. Second, the duration of underlying heart disease or the times of administration by IHD or HF were unknown. These parameters might associate the anxiety and depression. Third, we found the differences of associations with depression and anxiety and physical performances with underlying heart disease, however, the scores of HADS-anxiety and -depression were similar with underlying heart disease. Other mental status which cannot be detected by HADS might affect physical performances. Fourth, the patients who could not perform CPET were excluded. Some selection bias might affect the results in this study. Fifth, the effects of psychological intervention combined with exercise-based CR will need to be investigated further.

## 5. Conclusions

Depression and anxiety involved poor overall functional mobility and walking distance in CR outpatients. Especially in IHD patients, depression and anxiety involved high fat mass in body composition and low exercise tolerance and ventilator equivalents in CPET. To evaluate accurate physical performance, it is necessary to consider psychological condition. Our research showed the possibility that improvement of psychological condition causes improvement of physical performance. Further prospective interventional study is needed.

## Figures and Tables

**Table 1:** The differences of patient background between normal HADS group and high HADS group in whole patients, IHD patients, and HF patients.

	Whole Patients		*p* Value	IHD Patients		*p* Value	HF Patients		*p* Value
	Normal HADS	High HADS		Normal HADS	High HADS		Normal HADS	High HADS	
	*n* = 68	*n* = 32		*n* = 23	*n* = 15		*n* = 27	*n* = 7	
Age, years	70.2 (10.2)	71.1 (8.5)	0.56	68.7 (11.9)	70.1 (8.0)	0.71	70.9 (8.7)	70.6 (10.0)	0.42
Male, n(%)	48 (70.6)	25 (78.1)	0.43	21 (91.3)	15 (80.0)	0.31	13 (48.1)	4 (57.1)	0.67
BMI, kg/m^2^	23.2 (21.4–25.8)	23.7 (22.1–27.9)	0.14	23.3 (21.8–24.7)	23.9 (22.7–28.8)	0.07	23.4 (20.6–26.7)	22.4 (21.4–29.7)	0.68
LVEF, %	58.8 (50.4–67.2)	63.6 (43.8–69.0)	0.73	57.9 (53.2–66.1)	63.3 (46.9–70.2)	0.80	56.9 (44.5–67.1)	68.8 (36.7–73.6)	0.74
Frequency of CR, n(%)	1 (1–2)	1 (1–2)	0.24	1 (1–2)	1 (1–2)	0.06	2 (1–2)	1 (1–2)	0.11
Hypertension, n(%)	52 (76.5)	29 (90.6)	0.09	19 (82.6)	14 (93.3)	0.34	20 (74.1)	6 (85.7)	0.52
Diabetes mellitus, n(%)	22 (32.4)	18 (56.3)	0.02	10 (43.5)	9 (60.0)	0.32	7 (25.9)	1 (14.3)	0.52
Dyslipidemia, n(%)	54 (79.4)	31 (96.9)	0.02	22 (95.7)	15 (100)	0.41	15 (55.6)	6 (85.7)	0.14
CKD, n(%)	42 (61.8)	22 (68.8)	0.50	9 (39.1)	10 (66.7)	0.10	22 (81.5)	2 (28.6)	0.01
COPD, n(%)	2 (2.9)	1 (3.1)	0.96	0	0		2 (7.4)	0	0.46
The other pulmonary disease, n(%)	5 (7.4)	3 (9.4)	0.73	1 (4.3)	1 (6.7)	0.75	4 (14.8)	0	0.28
Psychiatric disorder, n(%)	2 (2.9)	3 (9.4)	0.17	1 (4.3)	2 (13.3)	0.32	1 (3.7)	0	0.61
Cardiovascular disease									
IHD, n(%)	35 (51.5)	23 (71.9)	0.05	23 (100)	15 (100)		0	0	
Heart failure, n(%)	39 (57.4)	15 (46.9)	0.33	0	0		27 (100)	7 (100)	
Valvular disease, n(%)	14 (20.6)	3 (9.4)	0.16	2 (8.7)	0	0.24	8 (29.6)	2 (28.6)	0.96
Cardiomyopathy, n(%)	13 (19.1)	2 (6.3)	0.09	0	0		12 (44.4)	1 (14.3)	0.14
Vascular disease, n(%)	7 (10.3)	2 (6.3)	0.51	1 (4.3)	0	0.41	4 (14.8)	0	0.28
Treatment									
PCI, n(%)	30 (44.1)	19 (59.4)	0.15	20 (87.0)	13 (86.7)	0.98	0	0	
CABG, n(%)	4 (5.9)	5 (15.6)	0.11	3 (13.0)	3 (20.0)	0.57	0	0	
Valve Operation, n(%)	11 (16.2)	2 (6.3)	0.17	1 (4.3)	0	0.41	7 (25.9)	1 (14.3)	0.52
ICD/CRT, n(%)	6 (8.8)	3 (9.4)	0.93	0	1 (6.7)	0.21	6 (22.2)	1 (14.3)	0.64

IHD: ischemic heart disease, HF: heart failure, HADS: hospital anxiety and depression scale, BMI: body mass index, LVEF: left ventricular ejection fraction, CR: cardiac rehabilitation, CKD: chronic kidney disease, COPD: chronic obstructive pulmonary disease, IHD, PCI: percutaneous coronary intervention, CABG: coronary artery bypass grafting, ICD: implantable cardioverter-defibrillator, CRT: cardiac resynchronization therapy.

**Table 2:** The differences of body composition between normal HADS group and high HADS group in whole patients, IHD patients, and HF patients.

	Whole Patients		*p* Value	IHD Patients		*p* Value	HF Patients		*p* Value
	Normal HADS	High HADS		Normal HADS	High HADS		Normal HADS	High HADS	
	*n* = 68	*n* = 32		*n* = 23	*n* = 15		*n* = 27	*n* = 7	
Body weight, kg	63.3 (55.2–67.1)	62.9 (56.2–71.4)	0.29	64.9 (57.8–67.5)	66.9 (62.5–76.6)	0.21	60.4 (12.6)	59.8 (14.6)	0.92
Lean body mass, kg	49.3 (39.6–52.3)	46.5 (40.8–51.3)	0.47	47.4 (42.4–51.2)	48.1 (37.5–49.1)	0.37	39.6 (8.7)	39.9 (7.5)	0.95
Body fat mass, kg	14.8 (11.7–17.6)	15.8 (13.2–23.8)	0.15	14.5 (11.7–17.5)	19.3 (15.0–24.8)	0.003	14.6 (11.2–20.1)	13.7 (9.7–15.0)	0.30
Percent body fat, %	24.4 (7.5)	27.7 (8.6)	0.07	22.2 (6.8)	30.5 (7.3)	0.001	27.2 (8.1)	22.8 (8.5)	0.34
Dry lean mass, kg	44.3 (7.2)	44.8 (10.8)	0.83	50.0 (44.7–54.0)	50.2 (39.6–51.4)	0.24	41.8 (9.2)	42.1 (7.9)	0.98
Total body water, L	32.7 (5.1)	32.3 (6.2)	0.75	34.1 (4.6)	33.1 (7.1)	0.61	29.9 (6.1)	29.3 (4.9)	0.85
Estimated bone mass, kg	2.60 (2.23–2.73)	2.45 (2.15–2.70)	0.30	2.60 (2.40–2.90)	2.65 (2.10–2.70)	0.20	2.20 (1.90–2.70)	2.35 (1.90–2.55)	0.82
Basal metabolic rate, kcal	1282 (187)	1256 (200)	0.56	1339 (161)	1292 (211)	0.45	1169 (230)	1139 (186)	0.82
Trunk lean mass, kg	25.5 (22.2–27.1)	24.8 (21.8–26.2)	0.19	25.9 (24.0–28.3)	25.5 (19.3–26.5)	0.09	20.4 (19.2–25.5)	24.2 (19.9–25.7)	0.75
Right arm lean mass, kg	2.25 (0.47)	2.28 (0.48)	0.76	2.46 (0.40)	2.38 (0.53)	0.62	1.70 (1.50–2.40)	2.25 (1.75–2.30)	0.98
Left arm lean mass, kg	2.30 (1.83–2.53)	2.15 (2.00–2.55)	0.95	2.37 (0.38)	2.30 (0.47)	0.60	1.90 (0.57)	1.98 (0.46)	0.81
Right leg lean mass, kg	7.70 (1.58)	7.67 (1.71)	0.95	8.17 (1.25)	8.02 (1.76)	0.77	6.88 (1.93)	6.68 (1.36)	0.85
Left leg lean mass, kg	7.54 (1.55)	7.55 (1.72)	0.98	7.94 (1.26)	7.88 (1.78)	0.85	6.75 (1.92)	6.55 (1.25)	0.85
Trunk fat mass, kg	9.00 (6.20–10.48)	9.35 (8.05–12.95)	0.15	8.70 (6.30–10.30)	11.65 (8.70–15.60)	0.005	8.70 (5.90–10.70)	8.20 (5.80–8.70)	0.39
Right arm fat mass, kg	0.60 (0.45–0.75)	0.65 (0.50–0.90)	0.21	0.60 (0.40–0.70)	0.80 (0.60–1.00)	0.003	0.60 (0.40–0.80)	0.55 (0.35–0.65)	0.44
Left arm fat mass, kg	0.60 (0.45–0.75)	0.70 (0.55–0.90)	0.10	0.60 (0.50–0.80)	0.80 (0.70–1.10)	0.002	0.60 (0.50–0.80)	0.55 (0.40–0.65)	0.41
Right leg fat mass, kg	2.45 (1.88–2.95)	2.65 (2.05–3.50)	0.20	2.40 (1.80–2.80)	3.00 (2.70–4.30)	0.002	2.50 (2.30–3.40)	2.30 (1.65–2.55)	0.23
Left leg fat mass, kg	2.45 (1.90–2.93)	2.65 (2.10–3.45)	0.13	2.50 (1.80–2.90)	3.10 (2.60–4.30)	0.001	2.50 (2.30–3.30)	2.30 (1.70–2.55)	0.26

IHD: ischemic heart disease, HF: heart failure, HADS: hospital anxiety and depression scale.

**Table 3:** The differences of several physical activity tests between normal HADS group and high HADS group in whole patients, IHD patients, and HF patients.

	Whole Patients		*p* Value	IHD Patients		*p* Value	HF Patients		*p* Value
	Normal HADS	High HADS		Normal HADS	High HADS		Normal HADS	High HADS	
	*n* = 68	*n* = 32		*n* = 23	*n* = 15		*n* = 27	*n* = 7	
Timed Up and Go Test, sec	6.75 (5.95–7.70)	7.52 (6.64–9.09)	0.01	6.25 (6.00–7.60)	7.20 (6.60–9.00)	0.07	6.90 (6.00–8.50)	6.90 (6.00–9.60)	0.77
Right-legged ST test, sec	35.9 (18.4–60.0)	26.5 (11.2–60.0)	0.12	60.0 (23.4–60.0)	39.2 (7.9–60.0)	0.33	27.4 (14.1–60.0)	18.6 (12.3–47.2)	0.51
Left-legged ST test, sec	30.8 (10.6–60.0)	28.4 (6.0–60.0)	0.27	47.1 (10.6–60.0)	43.5 (11.2–60.0)	0.65	26.3 (7.7–60.0)	21.8 (6.0–53.3)	0.75
SPPB, points	12.0 (12.0–12.0)	12.0 (11.5–12.0)	0.61	12.0 (12.0–12.0)	12.0 (12.0–12.0)	0.83	12.0 (12.0–12.0)	12.0 (10.0–12.0)	0.55

IHD: ischemic heart disease, HF: heart failure, HADS: hospital anxiety and depression scale, LS: locomotive syndrome, GLFS-25: 25-question Geriatric Locomotive Function Scale, ST test: standing time with eyes open test, SPPB: short physical performance battery.

**Table 4:** The differences of 2 min walking test between normal HADS group and high HADS group in whole patients, IHD patients, and HF patients.

	Whole Patients		*p* Value	IHD Patients		*p* Value	HF Patients		*p* Value
	Normal HADS	High HADS		Normal HADS	High HADS		Normal HADS	High HADS	
	*n* = 68	*n* = 32		*n* = 23	*n* = 15		*n* = 27	*n* = 7	
Distance, m	202 (185–216)	186 (162–208)	0.01	211 (200–238)	194 (178–210)	0.05	191 (26)	189 (24)	0.83
RPE, points									
Baseline	11 (9–12)	11 (11–11)	0.96	11 (8–11)	11 (10–11)	0.97	11 (11–12)	11 (11–12)	0.63
After 1 min	11 (11–13)	13 (12–14)	0.26	11 (11–12)	11 (11–12)	0.68	12 (11–13)	13 (11–16)	0.22
After 2 min	13 (11–13)	13 (12–14)	0.22	13 (11–13)	13 (12–13)	0.92	13 (11–14)	13 (12–17)	0.38
SpO_2_, %									
Baseline	97 (97–98)	97 (96–98)	0.28	97 (97–98)	97 (96–98)	0.53	97 (97–98)	96 (96–98)	0.52
After 1 min	97 (96–97)	96 (96–97)	0.15	97 (95–97)	97 (96–97)	0.75	97 (96–98)	96 (94–97)	0.09
After 2 min	96 (95–97)	96 (94–96)	0.09	96 (94–97)	96 (94–97)	0.53	96 (95–97)	94 (92–95)	0.01
HR, /min									
Baseline	76 (69–86)	82 (70–96)	0.38	80.2 (13.7)	81.5 (16.7)	0.80	73.3 (11.8)	81.1 (17.1)	0.16
After 1 min	91.0 (16.5)	91.5 (14.5)	0.92	94.6 (17.3)	92.5 (16.6)	0.71	85.1 (16.1)	91.4 (9.7)	0.33
After 2 min	98.0 (17.1)	96.2 (14.8)	0.56	102.9 (18.3)	96.8 (16.3)	0.31	93.2 (17.3)	95.0 (9.8)	0.80

IHD: ischemic heart disease, HF: heart failure, HADS: hospital anxiety and depression scale, SpO2: saturation of percutaneous oxygen, RPE: rate of perceived exertion.
